# Do male and female soccer players differ in helping? A study on prosocial behavior among young players

**DOI:** 10.1371/journal.pone.0209168

**Published:** 2018-12-17

**Authors:** Paul A. M. Van Lange, Zoi Manesi, Robert W. J. Meershoek, Mingliang Yuan, Mengchen Dong, Niels J. Van Doesum

**Affiliations:** 1 Institute for Brain and Behavior Amsterdam, Department of Experimental and Applied Psychology, Faculty of Behavioural and Movement Sciences, VU Amsterdam, Amsterdam, The Netherlands; 2 School of Social Sciences, Singapore Management University, Singapore, Singapore; 3 Institute of Developmental Psychology, Beijing Normal University, Beijing, China; 4 Social and Organizational Psychology, Faculty of Social and Behavioural Sciences, Leiden University, Leiden, The Netherlands; University of New Mexico, UNITED STATES

## Abstract

Acting prosocially can be quite challenging in one of the most salient intergroup contexts in contemporary society: Soccer. When winning is the ultimate goal, balancing self-interest with helping a fellow player in distress can be a tough decision; yet it happens. To date, we know little about what motivates soccer players to offer such help in the heat of the game. We propose that sex and what is at stake will matter in such prosocial dilemma situations. A pilot study (*N* = 107) indicated that female players may be more likely to help than male players, but this difference was only observed when the players are close to scoring position rather than far away from the goal (midfield). The main study (*N* = 366) finds that young soccer players show elevated inclinations to help in low-stakes situations, for example when their team is winning or when the outcome of the game seems pretty much decided. Contrariwise, helping intentions decline in high-stakes situations, for example when one’s own team is losing, when one is close to a scoring position in the offense (rather than at the midfield), or when the outcome of the game is still uncertain. Furthermore, female players show somewhat greater inclinations to help than their male counterparts. The current data point at some differences for male and female soccer players, albeit small in effect size. In contrast, we conclude that especially quick cost-benefit judgments regarding the stakes can play a major role in decisions to help or not to help another player on the soccer field.

## Introduction

Acting prosocially in a competitive intergroup context can be challenging. However, soccer is a highly competitive sport in which acts of kindness still occur. Despite substantial research on prosocial behavior (e.g., [1,2]), it is still a puzzle what motivates individuals to offer help in highly competitive contexts, such as a soccer game. Clearly, in soccer, the potential costs for offering help can change quickly and substantially depending on whether the player is in a goal scoring position (or midfield) or whether one’s own team is in a winning (or losing) position. Thus, focusing on helping in soccer allows us to better understand the circumstances under which prosocial behavior occurs in highly competitive intergroup settings.

One of the most significant prosocial acts during a soccer game is the decision to actually stop playing to help another player who fell on the ground and may have been injured. This is because deciding to offer help usually involves a trade-off between pursuing victory and assisting someone in need: There are typically few opportunities to take the lead in the game, and forgoing such opportunities for the sake of others can be too costly for the game’s outcome. But the play is regularly and intentionally interrupted by players, for example by kicking the ball out of the playing field or by offering a hand to a player who has been knocked to the ground.

The major purpose of the present research is to examine potential differences between male and female soccer players, as well as various differences in the game itself, on the decision to stop playing to check on a fellow player who is lying on the ground. Therefore, the key questions of the present research are: Do young female and male soccer players differ in inclinations to help? And does the inclination to help depend on the score of the game, such as whether one’s team is ahead or behind in score, and whether the game’s outcome is decided or uncertain? The latter variables are discussed in terms of “stakes” and how costly it is to offer help.

Although soccer has traditionally been a male-dominated sport at a global level (the USA being the well-known exception), female responses in such dilemma situations cannot be neglected, especially because female participation in soccer is rapidly increasing. Since 2012, the number of national academies for females has doubled whereas the number of registered female soccer players has exceeded one million [3]. Also, games at various levels (from local competitions to world championships) get more and more media attention. But despite the enormous growth in popularity of female soccer, we know little about female (versus male) responses in such dilemma situations during a soccer game.

Beyond the focus on sex differences in soccer, the present research extends past research in at least two theoretically meaningful ways. First, the present research focuses on prosocial behavior in a soccer context. This topic has also received little attention, even though there is increasing attention for research on prosociality in situations characterized by an intergroup conflict–the conflict between the interests of the own group and those of another group [4,5]. These intergroup conflicts pose interesting dilemmas in which parochial forms of cooperation can undermine collective interest. Specifically, helping members of the own group is sometimes conflicting with the broader, collective interests involving the interests of both groups. For example, encouraging unfair behavior (e.g., supporting a linesmen from the own club to favor the own team in signaling offside) might help the own team but it undermines the overall spirit of fairness in soccer–and to win and do so in a fair manner. Second, past research on cooperation and prosocial behavior has yielded a wealth of knowledge about the role of cognitive and affective processes, such as attitudes and social norms, religiosity, empathy, and evolved mechanisms such as kinship, reciprocity, and reputation [6–8].

The present research complements this (often lab-based) research by examining prosocial behavior in a real world context where attitudes and social norms are embedded in a long history of one of the most intense forms of civilized intergroup conflict (soccer), where empathy may still guide behavior, and where reciprocity and especially reputation may matter. But the intriguing aspect is that reputation and norms are not always clear–“should I stop playing when I see a player from the other team falling down?” In that sense, the intergroup context of soccer provides an ecologically meaningful context of intergroup conflict where a multifaceted package of motives, cognitions, and emotions might be activated, even under high levels of uncertainty and urgency.

### High versus low stakes

In their classic Good Samaritan study, Darley and Batson [9] showed that people are less likely to go out of their way to help someone slumped by the side of the road when they are in a hurry: The inclination to stop to help this person was lower under time constraints as compared to situations in which there was no time pressure (see also [10]). This finding suggests that in many daily situations in which precious resources are limited or the stakes are high, prosocial behavior tends to decrease. Seeing someone lying on the ground is a rather uncommon event in real life, but very common in soccer: Often, players stumble, fall, and sometimes get severely injured. It also concerns decision-making under uncertainty, because one needs to quickly judge whether the player just falls with low risk of injury, or falls with a high risk of injury. Using a situation similar to the original Good Samaritan study, soccer provides a context where help is frequently needed and the dilemma whether to check on a person who fell on the ground is often encountered.

In line with Darley and Batson’s work [9], other studies show that low (rather than high) stakes can increase prosocial behavior [11,12]. For instance, people seem to work harder for charity than for themselves when financial incentives are low, but they prefer the opposite when the incentives are raised [12]. Thus, the inclination to benefit others instead of oneself appears to be greater when the decision to act prosocially involves relatively low costs (or potential costs) for oneself.

Although there is some research on prosocial behavior in soccer and sports in general (e.g., [13,14]), most of the studies to date have focused on the role of personality and individual differences (for a recent review, see [15]). However, we know very little about whether helping behavior during the game can vary according to characteristics of *the game itself*–especially the stakes (or how psychologically costly it is to offer help). Here we approach stakes dilemmas as follows: Stakes are high (i.e., it is psychologically costly to offer help) when the player is virtually in goal scoring position, one’s team is behind in score, or the game’s outcome is uncertain (the score difference is small). In contrast, stakes are low when the player is midfield, one’s team is ahead in score, or the game’s outcome seems to be decided (the score difference is large).

Based on the above evidence, we propose that soccer players will be more inclined to check on a player lying on the ground when the stakes are low rather than high. Furthermore, aiming to replicate previous research [16–18], we predict that soccer players will show greater helping intentions toward teammates than to opponents. Acting prosocially toward a teammate (rather than an opponent) appears to be more habitual as people have a strong tendency to favor the own group over other groups, a tendency that is likely to be more pronounced in a competitive context [19]. This can be attributed especially to “ingroup love” (i.e., positive affect toward teammates) including strong ingroup ties (i.e., perception of similarity and bonding with teammates), which, in turn, increase prosocial behavior toward teammates [16].

### Sex differences in helping

Although some studies suggest that males and females behave differently during sports (e.g., [18–22]) it is not entirely clear how strong, meaningful, or robust sex differences are in prosocial behavior in the context of sports. For example, we know that as compared to males, female athletes tend to show a greater tendency to adhere to fair play norms by respecting the rules of the game and behaving cooperatively during competition [18]. As compared to males, females tend to show lower tolerance toward, and tendency to engage in, antisocial behaviors during competition, such as injurious behavior, intimidation of opponents and cheating behavior [14,18,20]. Thus, females appear to show a greater inclination to follow cooperative heuristics and to avoid antisocial behavior. However, we do not know whether there are also sex differences in helping behavior. More specifically, are female soccer players more inclined to check on a fellow player lying on the ground as compared to their male counterparts?

Whether to stop or continue playing may be guided specific social value orientations, which formally are described as preferences for maximizing own outcomes, a team’s outcomes, a relative advantage over the other team’s outcomes, and a concern with equality in outcomes [23,24]. Interestingly, research has uncovered interesting developmental differences in social value orientations. Specifically, research by Knight and Chao has demonstrated that girls and boys do not necessarily differ in terms of individualism, but that they differ in terms of egalitarianism and competitive orientation (sometimes also called rivalry or superiority, 23). Using various decision-making tasks, girls tend to be more egalitarian in orientation than boys: Girls favor more strongly equality so that the self and other are equally well-off (“fair share”). In contrast, boys are more prone to compete with others, seeking to obtain greater outcomes than others [23,25].

Complementary research suggests that girls tend to show greater prosociality in a broader sense, as expressed by empathic concern, nurturance, caring for others, and tending to the needs of other people [26,27]. This sex difference is mainly evident in close and long-term relationships (rather than interactions with strangers and contexts that involve danger) and tends to increase with age [28–31]. This can be due to the fact that, as compared to boys, girls to assign greater priority to self-transcendence values (universalism, benevolence), which emphasize concern for the welfare of others [32]. These general differences in social value orientation suggest that girls might be more prone to stop playing when a player falls, so as to do the same that one hopes another player would do for you. And perhaps even more importantly, if boys are generally more competitive in orientation, the desire to win may overshadow a concern with a player who falls on the ground.

While the scientific evidence would generally suggest that women are more likely to help than men, the question is whether such differences may also be uncovered on the soccer field. There are at least three reasons why such differences may be small or virtually absent. First, sex differences in helping and prosociality have been observed but tend to be small in magnitude [33,34]. Second, sex differences in helping among adolescents may be small because most people in those age groups tend to be individualistic rather than prosocial in orientation [34,35]. And third, most importantly, the soccer context is a “strong situation” (e.g., [36]) characterized by strong impulses and norms to compete [37]. It is plausible that the effects of context are so strong that they overshadow any differences between young men and women.

### Present research

This research is one of the first attempts to examine differences between young men and women on the soccer field. We used scenario methodology that we first tested in a pilot study. Although there are limitations to such a methodology, it does provide initial insight into the potential differences between young men and women in helping behavior on the soccer field. Moreover, this methodology allows us to examine the effects of contextual variations on self-reported helping. To manipulate the size of stakes in the main study, the scenario described a hypothetical situation in which one’s own team is in a winning (or losing) position and the score difference between one’s own team and the opposing team is large (or small). Furthermore, the fallen player was portrayed to be a teammate (or an opponent).

We hypothesize that the size of the stakes and the player’s sex will affect helping behavior on the soccer field. More specifically, soccer players are expected to show greater inclination to help a fallen player in low (rather than high) stakes dilemmas and, hence, when one’s own team is in a winning (rather than losing) position, or when the score difference is large (rather than small). Furthermore, we expect female (rather than male) players to show greater inclinations to help a player who has fallen on the ground. Further, we predict that players will show greater helping intentions toward teammates rather than opponents.

It needs to be noted that we focused specifically on young soccer players (aged between 9 and 19 years) in amateur soccer. We chose this group because we speculated that stakes would vary more strongly in this age (young rather than older) and level (amateur versus professional). Furthermore, this convenience sample allowed us to access comparably high numbers of male and female soccer players. Given that the present study is one of the first studies examining sex effects in soccer, we advanced no formal hypothesis as to how sex may interact with either the stakes of the game or the teammate versus opponent difference. Rather, we wanted to explore whether the stakes of the game and the differences between helping a teammate versus an opponent would be more pronounced for men than for women, or vice versa.

## Pilot study

### Materials and methods

#### Ethics statement

The studies were reviewed and approved by the Scientific and Ethical Review Board (VCWE) of the Faculty of Behavioral and Movement Sciences, VU Amsterdam. Participants provided electronic consent prior to taking the online survey (for the pilot study) or written consent prior to taking the paper-and-pencil survey (for the main study).

#### Participants

One hundred and seven (107) young Dutch soccer players (52 female; *M*_age_ = 14.27 years, *SD* = 2.35 years) completed an online survey. We recruited participants from local soccer clubs in different regions of the Netherlands. Initially, we contacted the directors of 13 clubs and asked them to distribute the survey among young soccer players. From the total of 13 clubs, four agreed to participate in this research and the trainers of the clubs sent the online survey link to the soccer players via email. Participation was voluntary but teams whose participation exceeded 50% were promised a small monetary reward (there was only one team that accomplished this goal and received 25 euros for drinks after a game).

#### Procedure, design, and measures

All participants filled out the online survey at home. After reading the informed consent form and agreeing to participate, the young soccer players read certain scenarios and answered questions related to helping during a soccer match. Next, they provided some demographic information, were debriefed and thanked. We employed a mixed design with sex as between-participants variable and scenarios as within-participants variable. We also controlled for age as a covariate.

We measured helping by examining the inclination to stop playing to check on another player who fell on the ground during a soccer match. The manipulation aimed to provide an initial test that helping might be affected by circumstances. In an effort to create an appropriate manipulation of stakes, the scenarios in this pilot study varied such that the position of the participant was either midfield or in goal scoring position. Helping when being midfield (as compared to goal scoring position) should be a low-stakes dilemma (as compared to high-stakes dilemma). Furthermore, we manipulated the team of the fallen player, such that the player was either a teammate or an opponent.

To cover a broad range of helping behavior, we assessed two complementary expressions of helping toward the fallen player: (a) the inclination to neglect the player and continue the game, and (b) the inclination to actually stop the game and check how the player was doing. Thus, using a 5-point scale (ranging from *definitely not* to *definitely yes*), participants rated the extent to which they were inclined to ignore the fallen player and continue playing (reverse scored), as well as the extent to which they were inclined to stop playing to see how the fallen player was doing. For a more complete and accurate evaluation of helping, we used the combined average between the two items (all *α*s were between 0.84 and 0.91). Higher scores indicated higher levels of the inclination to help.

### Results and discussion

We performed a 2 (Position: goal scoring, midfield) by 2 (Player: teammate, opponent) by 2 (Sex: male, female) mixed model ANOVA with Position and Player as within-participant factors, Sex as between-participants factor, and centered value of Age as covariate. Data revealed a significant main effect of Position, *F*(1, 104) = 71.202, *p* < .001, η_p_^2^ = .406, such that participants were more inclined to help when they were midfield (*M* = 3.27, *SD* = 0.97) rather than in a goal scoring position (*M* = 2.57, *SD* = 0.92). Furthermore, there was a significant main effect of Player, *F*(1, 104) = 35.682, *p* < .001, η_p_^2^ = .255, such that participants were more inclined to help teammates (*M* = 3.16, *SD* = 0.94) than opponents (*M* = 2.69, *SD* = 0.93). Against expectation, data revealed no significant main effect of Sex on helping (*p* = .476). However, we found a significant interaction between sex and position, *F*(1, 104) = 6.867, *p* = .010, η_p_^2^ = .062. Subsequent posthoc (LSD) pairwise comparisons showed that while female (*M* = 3.22, *SD* = 0.98) and male (*M* = 3.33, *SD* = 0.98) players did not differ in their willingness to help in a midfield position, *F*(1, 104) = 0.286, *p* = .594, females (*M* = 2.74, *SD* = 0.92) were more inclined to offer help than males (*M* = 2.41, *SD* = 0.92) in a goal scoring position, *F*(1, 104) = 3.551, *p* = .062, η_p_^2^ = .033. None of the main effect of Age (*p* = .840), or other two-way (all *p*-values > .089) or three-way interaction (all *p*-values > .252) was significant. Data and syntax are available as S1 Dataset.

Overall, the pilot study suggests that stakes matter in helping on the soccer field: Young soccer players indicated greater inclinations to help when the stakes were low (i.e., when being midfield rather than in goal scoring position). Furthermore, the inclination to help was greater when the fallen player was a teammate rather than an opponent. Thus, the pilot confirmed the validity of the method. Although marginally significant, females were more likely to help than males when they were in a goal scoring position (but not in a midfield position). Considering that effect sizes for sex differences in prosocial behavior tend to be small to moderate [31], having a small sample size might explain why we found no support for a general sex difference in helping behavior. In the main study, we aimed to address this issue by recruiting a larger sample.

## Main study

Aiming to increase the statistical power, in the main study, we visited four different soccer clubs in the Netherlands, anticipating that we could recruit at least 200 participants to be able to detect even small effect sizes. Furthermore, in view of the positive finding of the pilot experiment, the main study aimed to refine the manipulation of stakes and improve its precision. Here, we used the same methodology but we focused on variations in stakes that are linked to the score in the present moment (ahead versus behind in the game, small versus large score difference). More specifically, to vary the size of stakes, instead of focusing on midfield (versus goal scoring) position, the new scenarios focused on winning (versus losing) position and on large (versus small) score difference. To strengthen the importance of the decision, we amended the scenarios so that the incident with the fallen player appeared to take place five minutes before the end of the game. We also addressed the limitations of the previous online sample by visiting the players and collecting data at their local clubs.

### Materials and methods

#### Participants

Three hundred and seventy nine (379) young Dutch soccer players completed a paper-and-pencil survey. Of this initial sample, data of 13 participants were discarded due to incomplete responses. This yielded a final sample of 366 participants (157 female, 207 male, 2 unreported; *M*_age_ = 14.39 years, *SD* = 2.24 years, 4 unreported). As with the pilot experiment, the young soccer players were recruited in four local soccer clubs in different regions of the Netherlands. Participation was voluntary and each participant received a sports drink as compensation for completing the survey.

#### Procedure, design, and measures

All participants filled out the paper-and-pencil survey at their local soccer club. After reading the informed consent form, the young soccer players read several scenarios and answered items assessing helping on the soccer field. To conclude they answered some demographic questions, received compensation, and were debriefed and thanked. We again used a mixed design with sex as between-participants variable and scenarios as within-participants variable, and included players’ age as covariate.

The measure of helping was identical to that used in the pilot study with two differences related to the size of stakes: (a) position now indicated either winning or losing position, and (b) we introduced score difference as a new parameter (small versus large). The game was told to last about another 5 minutes, and the score difference was either small (2–1) or large (5–1). Also, in addition to these scores, the difference in favor of the own team (winning) or other team (losing) was highlighted by the terms “small” (e.g., in Dutch, “je staat krap voor”) or “large” (e.g., in Dutch, “je staat dik voor”) using language that seems common among young players in the context of soccer games. Similar to the pilot study, higher scores indicated higher levels of inclination to help (all *α*s ranged from 0.77 to 0.91).

### Results

We conducted a 2 (Position: winning, losing) by 2 (Score Difference: small, large) by 2 (Player: teammate, opponent) by 2 (Sex: male, female) mixed model ANOVA with Position, Score Difference and Player as within-participant factors, Sex as between-participants factor, and centered value of Age as covariate. The analysis yielded a significant main effect of Position, *F*(1, 358) = 75.304, *p* < .001, η_p_^2^ = .174, suggesting that participants were more inclined to help in a winning (*M* = 2.66, *SD* = 0.93) than in a losing position (*M* = 2.34, *SD* = 0.89). Score Difference also had a main effect on helping, *F*(1, 358) = 256.028, *p* < .001, η_p_^2^ = .417), in the sense that participants were more inclined to help when the score difference was large (*M* = 2.86, *SD* = 1.03) rather than small (*M* = 2.14, *SD* = 0.86). Furthermore, there was a significant main effect of Player, *F*(1, 358) = 72.981, *p* < .001, η_p_^2^ = .169, such that participants were more inclined to help teammates (*M* = 2.65, *SD* = 0.91) than opponents (*M* = 2.35, *SD* = 0.90). This suggests that the size of stakes matter in the inclination to help.

We also found significant main effects of Age, *F*(1, 358) = 10.464, *p* = .001, η_p_^2^ = .028, and Sex, *F*(1, 358) = 11.860, *p* = .001, η_p_^2^ = .032. Younger players were more willing to help than the elder ones, and more importantly, females showed greater helping tendencies (*M* = 2.65, *SD* = 0.83) than males (*M* = 2.35, *SD* = 0.83). Although the effect size is small, this finding supports the hypothesis that women are more inclined than men to help others on the soccer field.

Furthermore, results yielded a significant two-way interaction between Position and Score Difference, *F*(1, 358) = 35.231, *p* < .001, η_p_^2^ = .090. Subsequent posthoc (LSD) pairwise comparisons showed that compared to when the score difference was low (*M*_winning_ = 2.21, *SD* = 0.97; *M*_losing_ = 2.07, *SD* = 0.91), when the score difference was high participants were more willing to help in winning position (*M* = 3.11, *SD* = 1.16), *F*(1, 358) = 274.564, *p* < .001, η_p_^2^ = .434, than in losing position (*M* = 2.62, *SD* = 1.14), *F*(1, 358) = 96.627, *p* < .001, η_p_^2^ = .213. Moreover, this two-way interaction was qualified by participants’ age, *F*(1, 358) = 13.513, *p* < .001, η_p_^2^ = .036. Specifically, the interaction between Position and Score Difference was significant for younger (-1 *SD*), *F*(1, 358) = 46.507, *p* < .001, η_p_^2^ = .115, but not older (+1 *SD*), *F*(1, 358) = 2.666, *p* = .103, participants. No other interaction effect was significant (all *p*-values > .079). Data and syntax are available as S1 Dataset.

Overall, the most novel finding from our main study concerns the sex differences in helping on the soccer field. Furthermore, this study replicates and extends findings from the pilot study by showing that the size of stakes (related to the team’s position in the game and the score difference) can affect the inclination to help. Also, the inclination to help was greater when the fallen player was a teammate rather than an opponent. And finally, helping was most likely when the younger players were in a comfortable position of winning with a large score difference.

## Discussion

The present research sheds light on the circumstances under which helping behavior on the soccer field emerges. Both the main and the pilot study demonstrated that young soccer players were more inclined to stop playing to check on a fellow player when the stakes in the game were low rather than high. Perhaps younger (amateur) players do not take the game as seriously as older (amateur) players, and therefore are more likely to stop playing. Also, beginning players may be more easily distracted by unexpected events. Both tendencies may be stronger when they are in a comfortable position of winning by big numbers. Further, the main study revealed that helping was higher when one’s own team was in a winning rather than in a losing position, or the game’s outcome seemed decided rather than uncertain. Furthermore, helping was greater toward a teammate (rather than an opponent). Importantly, the main study revealed sex differences in helping: Females were more inclined to help than males.

Our results replicate and extend the classic findings of Darley and Batson [9] by showing that the size of stakes can affect helping intentions even in a competitive team sport environment. When the ultimate goal is to win the game, the decision whether or not to forgo what is in the best interest of one’s own team to help someone in need strongly depends on how costly this decision may be. From a sports science perspective, this finding suggests that helping intentions during competition are more flexible than previously assumed, because they are not solely affected by individual differences in variables such as autonomous motivation, prosociality, or moral disengagement (e.g., [13,15,38]), but also by important features of the situation, such as those linked to the stakes of the games. Thus, this research draws attention to the importance of factors related to the game itself in helping behavior during a sports competition. Clearly, more research is needed to replicate and extend these findings in different sports contexts, which may include other team sports or even comparisons between individual and team sports to illuminate whether the own team (the ingroup) might inhibit helping another player. The present research suggests that prosocial behavior on the playing field might vary substantially depending on continuous and dynamic changes in the game situation.

Broadly speaking, our findings are consistent with predictions from gain versus loss framing and prospect theory [39,40]; as with other choice dilemmas, here we find that losses loom larger than gains. This asymmetry in the importance of losses in relation to gains may further explain why soccer players were more inclined to offer help when their team was winning (instead of losing). Moreover, considering the certainty effect, it appears plausible that helping intentions increased when the degree of certainty (for winning the game, especially) was higher (i.e., the score difference was large rather than small).

Perhaps the most novel finding of the main study was a sex difference in helping. Extending prior research [15], our results indicate that female soccer players were more inclined to help as compared to their male counterparts. Although past studies have shown that females, more than males, tend to respect rules and avoid antisocial behavior in sports (e.g., [41]), here we provide support to the notion that there are sex differences in helping. In the introduction, we highlighted three general arguments why sex differences among young players in the context of soccer are likely to be small–past research has shown modest effect sizes, the young age, the strong situational context of soccer. In other words, although differences in empathy (and caring) between men and women, even as soccer players, are often stereotyped as being strong, but the scientific evidence is somewhat less strong.

How do we explain the sex difference? One possible explanation is empathy: As compared to males, females tend to express greater empathic concern and sensitivity to distress in others and this is evident in both sports and non-sports contexts (e.g., [41–43]). This concern for the well-being of fellow players could be even greater among females (as compared to males) because the risks for them appear to be higher: For instance, females appear less likely to engage in deceptive falling (diving) and more likely to sustain serious injuries during sports [44,45]. We should also acknowledge that the differences in empathy are somewhat overestimated in the present research, because our findings are based on scenario-methodology. Indeed, it is possible that this “explicit measurement” is to some degree affected by sex-related stereotypes, along with norms for how to behave, even on the soccer field [31,31,46].

As a second possibility, our findings may also be explained by the notion that, girls value equality and fairness more strongly than boys do, who tend to be more strongly orientated to rivalry and competition [23, 25]. Thus, our finding provides support to the idea that there is a distinct female psychology that accounts for increased helping behavior at the expense of oneself; it is argued that such female psychology has evolved because it promotes fitness interests and is possibly enhanced by gender socialization [28,47]. Indeed, this reasoning is consistent with the view that the gender-roles of men and women are different, even for “strong” and specific situations such as competitive ingroup-outgroup games such as amateur soccer.

Needless to say, the present findings present some of the first evidence for sex differences in helping in sports. The results should thus be interpreted with caution. One possible explanation for the absence of main effects for sex in the pilot study is that the sample size was relatively small compared to that of the main study. Future replication studies with high statistical power are required to more firmly establish the link between sex and helping in soccer (and sports in general, team sports as well as individual sports).

It needs to be noted that the sex differences in helping behavior might be specifically observed in young soccer players but less so (or not at all) in professional players. Considering the heightened participation of females in soccer and the constantly increasing professionalism of female soccer [3], it is likely that professional female and male soccer players show comparable levels of helping as the stakes in top competitive teams are, by default, high. More research involving both amateur and professional players is required to generalize or identify boundary conditions for the present finding.

It is important to underline that the observed sex differences in helping were relatively small in magnitude. Furthermore, next to the sex differences, there were certain sex similarities that cannot be overlooked. For instance, male and female players responded similarly to the manipulation of stakes: Men and women were equally prone to show heightened help when being in a winning (versus losing) position. Also, both men and women tended to show heightened help when differences in score were large (versus small). This suggests that the two sexes are equally affected by the dynamic game situation, as they both tend to adjust their prosocial inclinations in response to the size of stakes. One potential exception to this “rule” is that men may be more likely than women to continue playing–and not stop to see what happened–when being closer to scoring. Perhaps at some critical and specific moments, when the stakes in soccer are very high (including personal stakes of scoring himself or herself), feelings of empathy may be more likely to be reduced in men than in women. This intriguing issue clearly deserves future research to provide insight into the robustness and generality across different situations on the field (e.g., other critical situations), types of team sport (e.g., volleyball, field hockey), and types of player (e.g., adult players).

### Limitations and future directions

One limitation of the present research is that the findings are based on self-report measures. It is not argued that self-report intentions to help on the soccer field will always result in real-world helping behavior during the game. However, when taking the exploratory nature of this research into account, this work provides initial evidence that the size of stakes and the players’ sex can affect prosocial intentions during the game. Furthermore, the study design allowed us to a priori circumvent intervening variables that could potentially affect the results (e.g., inability to notice the fallen player). For instance, in high-stakes situations (e.g., when being in goal-scoring position), it is likely that players experience such elevated levels of adrenaline and excitement that they might not even notice the fallen player lying on the ground. The present studies allowed us to rule out such spurious relationships and arbitrary “noise” in the data. Nevertheless, future observational experiments could help confirm the present findings.

Second, the present research focused on a specific form of prosocial behavior during a soccer game and, therefore, the effects cannot yet be generalized to all forms of prosocial behavior in a sports competition. Whether to help a fallen player constitutes decision-making under uncertainty. It could be that a fall entails risk, but it is perhaps more likely that most falls are relatively free of any risk of injuries. Also, although females expressed greater inclinations to stop playing to help a fallen fellow player, it is likely that males express greater helping intentions in other types of prosocial dilemmas. This is because males, more than females, tend to engage in helping behavior that is heroic and chivalrous (rather than nurturant and caring, see [29]). Further research is required to evaluate the sex effect on acts of helping that are heroic and involve physical risks versus acts of helping that are nurturant in a sports competition (e.g., saving a fellow player’s life versus helping a fellow player get up).

Third, the two studies do not provide information on potential mechanisms underlying the effects of stakes and sex on helping. For example, although empathy is a likely explanation of the effects of sex [48,49], it is yet to be demonstrated whether the players were inclined to help because of heightened understanding of another person’s emotions and concern about their welfare. Furthermore, there could be alternative mechanisms driving the effects, such as expectations for rewards by one’s teammates or fear of sanctions. More specifically, helping a fellow player when the stakes are low might help obtain a positive social image and gain a reputation as a cooperator [8,50]. Contrariwise, helping another player when the stakes are high may be perceived as an act of weakness or even betrayal of one’s own team that could lead to experiencing sanctions by one’s teammates. We should also note that perhaps acts of helping are promoted (or undermined) when people feel and think in autonomous ways–in a manner independent of how own or other team players might evaluate such behavior [51, 52]. But if the evaluations of the own team members guide helping (or not) on the soccer field, it is also likely that some processes are culture-specific. Growing evidence suggests that some cultures are more oriented to ingroup favoritism and collectivist mindsets [53–55]. Future research could look more deeply into possible explanations.

### Conclusions

Acting prosocially during a competitive soccer game is a challenge. Yet, under the right circumstances, young soccer players are inclined to help a fellow player in distress at the expense of personal or team success. The present research showed that the stakes of the situation matter: When the stakes for personal and team success are low, the inclination to help increases; contrariwise, when the stakes for success and victory are high, the motivation to help tends to be lower. This finding suggests that in competitive situations like a soccer game, the cost of the prosocial act matters because “players appear to help when it doesn’t hurt.” Furthermore, we found that males and females respond differently to prosocial dilemmas on the soccer field: Female soccer players expressed greater helping intentions than their male counterparts. Being among the first to examine differences between men and women in soccer, the present study is, of course, in need of replication and the findings require further exploration to advance the literature on differences–and similarities–between men and women on the soccer field.

## Supporting information

S1 Dataset Dataset pilot study and dataset main study. (ZIP)
